# Environmental Pollution: Health Effects and Operational Implications for Pollutants Removal

**DOI:** 10.1155/2012/341637

**Published:** 2012-05-06

**Authors:** Roya Kelishadi

**Affiliations:** Faculty of Medicine and Child Health Promotion Research Center, Isfahan University of Medical Sciences, Isfahan 81676-36954, Iran

 Environmental pollution is reaching worrying proportions worldwide. Urbanization and industrialization along with economic development have led to increase in energy consumption and waste discharges. The global environmental pollution, including greenhouse gas emissions and acid deposition, as well as water pollution and waste management is considered as international public health problems, which should be investigated from multiple perspectives including social, economic, legislation, and environmental engineering systems, as well as lifestyle habits helping health promotion and strengthening environmental systems to resist contamination [1–3].

 Environmental pollutants have various adverse health effects from early life some of the most important harmful effects are perinatal disorders, infant mortality, respiratory disorders, allergy, malignancies, cardiovascular disorders, increase in stress oxidative, endothelial dysfunction, mental disorders, and various other harmful effects [4, 5]. Though, short-term effects of environmental pollutants are usually highlighted, wide range of hazards of air pollution from early life and their possible implication on chronic non-communicable diseases of adulthood should be underscored. Numerous studies have exposed that environmental particulate exposure has been linked to increased risk of morbidity and mortality from many diseases, organ disturbances, cancers, and other chronic diseases [6, 7]. Therefore it is time to take action and control the pollution. Otherwise, the waste products from consumption, heating, agriculture, mining, manufacturing, transportation, and other human activities will degrade the environment.

 Based on the strength of the scientific knowledge regarding the adverse health effects of environmental pollution and the magnitude of their public health impact, different kinds of interventions should be taken into account. In addition to industrial aspects, the public awareness should be increased in this regard. Likewise, health professionals have an exclusive competency to help for prevention and reduction of the harmful effects of environmental factors, this capacity should be underscored in their usual practice.

 This special issue is dedicated to increasing the depth of research across all areas of health effects of pollutants in air, water, and soil environments, as well as new techniques for their measurement and removal. The goal of the special issue is to familiarize the readership of the Journal of Environmental and Public Health with the potential for different aspects of environmental pollution. We expect this special issue would appeal to researchers, public health practitioners, and policymakers.

*Roya Kelishadi* *Roya Kelishadi*
